# Randomized Controlled Clinical Trial of Pediatric Pneumococcus and Hepatitis A Vaccinations With or Without a High-Dose Oral Vitamin A Supplement

**DOI:** 10.3390/biom15040540

**Published:** 2025-04-07

**Authors:** Nehali Patel, Sherri L. Surman, Bart G. Jones, Rhiannon R. Penkert, Karen Ringwald-Smith, Kim DeLuca, Julie Richardson, Ying Zheng, Li Tang, Julia L. Hurwitz

**Affiliations:** 1 Department of Infectious Diseases, St. Jude Children’s Research Hospital, Memphis, TN 38105, USA; nehali.patel@stjude.org (N.P.); sherri.surman@stjude.org (S.L.S.); bart.jones@stjude.org (B.G.J.); kimberly.deluca@stjude.org (K.D.); 2 Department of Chemistry and Biochemistry, Institute of Molecular Biology, University of Oregon, Eugene, OR 97403, USA; rpenkert@uoregon.edu; 3 Department of Clinical Nutrition, St. Jude Children’s Research Hospital, Memphis, TN 38105, USA; karen.smith@stjude.org; 4 Department of Pharmacy and Pharmaceutical Sciences, St. Jude Children’s Research Hospital, Memphis, TN 38105, USA; julie.richardson@stjude.org; 5 Department of Biostatistics, St. Jude Children’s Research Hospital, Memphis, TN 38105, USA; ying.zheng@stjude.org (Y.Z.); li.tang@stjude.org (L.T.)

**Keywords:** retinol, retinol binding protein, pneumococcus vaccine, hepatitis A vaccine

## Abstract

Previous studies have shown that high-dose vitamin supplements can improve vaccine-induced immune responses and pathogen protection in the context of vitamin deficiencies. To further elucidate the influence of vitamin supplements on immune responses toward pediatric vaccines, we performed a randomized controlled clinical trial (PCVIT) of 20 healthy children 1–4 years of age in Memphis, Tennessee. Study participants received a booster vaccine for pneumococcus and a primary vaccine for hepatitis A virus with or without a high-dose, oral, liquid supplement of 10,000 IU retinyl palmitate. We found that the children enrolled in PCVIT had higher baseline vitamin levels than previously described older children and adults living in Memphis. Only one child in PCVIT had a serum retinol level of less than 0.3 µg/mL. The children frequently consumed milk and baby foods that were likely vitamin-fortified, providing an explanation for the relatively high vitamin levels. Most children in PCVIT responded well to pneumococcus and hepatitis A vaccines by pathogen-specific antibody upregulation. The one child with a serum retinol level below 0.3 µg/mL did not receive a vitamin supplement and exhibited the lowest fold-change in antibody responses toward pneumococcal serotypes. A correlation matrix encompassing demographics, vitamin levels, vaccine-induced immune responses, C-reactive protein, and total serum immunoglobulin isotypes, including IgG2 and IgA, identified variables associated with vaccination outcomes. Perhaps because children were predominantly retinol-sufficient at baseline, the high-dose vitamin A supplement exhibited no benefit to vaccine-induced immune responses. In fact, when vitamin supplemented and vitamin unsupplemented groups were compared among participants with the highest baseline retinol levels, there was a trend toward weaker vaccine-induced immune responses in the vitamin supplemented group. Results encourage the performance of larger clinical studies before high-dose vitamin supplements are recommended for populations that are otherwise vitamin-replete.

## 1. Introduction

Vitamin A is an essential nutrient acquired from the diet. Retinyl esters are generally stored in the liver, while retinol is frequently bound by retinol binding protein (RBP) in the circulatory system. Retinol dehydrogenase converts retinol to retinal and, in select tissues, retinal dehydrogenase converts retinal to retinoic acid (RA) [1,2,3]. Vitamin A has numerous mechanisms of action, including the transmission of cell signals when the retinol-RBP complex (holo-RBP) interacts with STRA6 at the mammalian cell surface [2,4,5]. In the nucleus, RA is a ligand for transcription factors including the retinoic acid receptors (RARs) and the peroxisome proliferator-activated receptors (PPARs). RA thereby influences gene expression in a plethora of cells, including B cells, T cells, dendritic cells, and epithelial cells [2,6,7,8,9,10,11]. Processes, including cell migration, cell attachment, and cytokine expression, are all affected [3,12,13,14,15,16,17,18,19]. DNA sequences that favor RAR binding, termed retinoic acid response elements (RARE), are located not only in gene promoters and known regulatory regions, but in switch sites central to gene rearrangements in the immunoglobulin heavy chain locus [8,20,21]. Thus, vitamin A may influence immunoglobulin class switch recombination as well as cytokine secretion to instruct immunoglobulin isotype expression patterns [1,19,22,23,24].

The association of vitamin A with the prevention and control of infectious diseases has been well documented, and in a number of pre-clinical and clinical studies, vitamin A supplements have improved vaccine-induced immune responses and pathogen protection [22,25,26,27,28,29,30,31,32,33,34]. Particularly noteworthy has been the use of vitamin A supplements in low-income countries to improve immunity and protection from the measles virus [26]. The World Health Organization (WHO) currently recommends a dose of 200,000 IU for children aged 12–59 months, given every 4 to 6 months in populations with vitamin A deficiencies [35,36,37,38].

While there have been recent calls to implement high-dose vitamin supplementation programs in the United States [39], the benefit of such programs in populations with replete vitamin status remains unclear. High-dose vitamin supplements are often, but not always beneficial [40,41,42]. Semba et al., for example [43], showed that vitamin A supplements dampened the immune response toward the measles virus in one population. As a second example, Bresee et al. showed that pediatric patients in the United States who were vitamin A supplemented upon hospitalization with respiratory syncytial virus (RSV) fared worse than unsupplemented controls [44]. As a third example, in a clinical study of vitamin A and D supplementation at the time of influenza vaccination in children 2–8 years of age, the supplements associated with improved immune responses in participants, but only when participants had relatively low levels of vitamins A and D at baseline.

To further elucidate the effects of vitamin supplementation on immune responses to pediatric vaccines, we performed a small study (PCVIT) in Memphis, TN, of healthy children 1–4 years of age. Participants received a booster pneumococcus vaccine and a hepatitis A vaccine with or without an oral supplement of 10,000 IU retinyl palmitate.

## 2. Materials and Methods

### 2.1. Study Design

The PCVIT study (IRB protocol #19-0064) was a randomized controlled clinical trial of antibody responses to vaccines administered with and without a high-dose vitamin A supplement, conducted at St. Jude Children’s Research Hospital in Memphis, TN. Initial IRB approval was on 3/19/19. The study was paused in 2020 during the COVID-19 pandemic and was resumed in 2023. There were 20 evaluable participants enrolled.

All legal guardians of enrolled participants signed informed consent on the screening visit. Informed consent followed institutional standard practices for research consents. Time was given for the guardian to ask questions, and all participation was voluntary. A copy of the consent was given to the legal guardian, and a copy was uploaded into the electronic medical record.

Vaccines were commercially approved, including the pneumococcus vaccine (Prevnar) given as a booster dose and the hepatitis A vaccine (HAVRIX) given as a priming dose. A change from Prevnar 13 to Prevnar 20 occurred in the year 2023 based on recommendations from the Centers for Disease Control and Prevention’s Advisory Committee on Immunization Practices. The thirteen pneumococcal serotypes represented in Prevnar 13 were 1, 3, 4, 5, 6A, 6B, 7F, 9V, 14, 18C, 19A, 19F, and 23F. The 20 pneumococcal serotypes represented in Prevnar 20 were 1, 3, 4, 5, 6A, 6B, 7F, 8, 9V, 10A, 11A, 12F, 14, 15B, 18C, 19A, 19F, 22F, 23F, and 33F. The primary objective of the PCVIT study was to evaluate antibody responses after vaccination within two cohorts, one that had received an oral vitamin A supplement of 10,000 IU retinyl palmitate at the time of vaccination and one that had not. The intervention was randomized equally, 1:1 for the two cohorts.

Healthy children 1–4 years of age who were fully weaned from formula feeding or breast milk for at least four weeks, who had previously received at least two doses of the pneumococcus vaccine, and who had not yet received a hepatitis A vaccine were eligible for enrollment. Exclusion criteria included a previous life-threatening allergy to a childhood vaccination, use of investigational or immunosuppressive medications, study-unrelated vitamin supplementation during the study period, evidence of developmental delay, evolving neurological disorders during the study period, current use of antibiotics or antivirals, and any history of diabetes, cardiac, kidney, or chronic respiratory conditions. Children were screened to exclude any febrile illness within three days of vaccination.

Vitamin A was the only vitamin given in this clinical trial. Vitamin A palmitate was compounded by Regal PharmaLab into a 10,000 IU/1 mL oral solution (~1 mL safflower oil edible USP with 2 microliters tangerine oil, natural flavor) per dose. Compounding followed U.S. Pharmacopeial Convention (USP) 795 compounding standards for pharmaceutical compounding of nonsterile preparations. The supplement dose was selected to exceed typical daily vitamin doses without adverse effects [45,46].

Blood taken at the screening visit defined retinol binding protein (RBP) and vitamin D levels. RBP was tested with a Human RBP4 quantikine ELISA kit (R&D systems, Minneapolis, MN, USA). RBP is a standard, albeit not exclusive carrier of retinol, often used as a surrogate for retinol levels with an approximate, predicted, 1:1 molar ratio [47,48]. Vitamin D was assayed using the Roche Elecsys Vitamin D assay, either Gen 2 (prior to October 2022) or Gen 3.

Participants returned to the clinic on the day of enrollment (Day 0), approximately one week after the screening visit, for vaccinations. After eligibility was confirmed, the participants were randomized to receive vaccines with or without the vitamin A supplement using an internal St. Jude randomization system and a stratified permuted block method based on serum RBP levels (<22,000 or ≥22,000 ng/mL) and serum vitamin D levels (<30 or ≥30 ng/mL), yielding four strata. Stratification was performed to ensure that participants had similar baseline vitamin levels in vitamin supplemented and vitamin unsupplemented groups [48]. Given that vitamins A and D may be cross-regulated and that baseline vitamin levels may affect the outcome of vitamin supplementation [8,9,10,21,28,34,49], both RBP [48] and vitamin D levels were used for stratification [10,45,46,50,51,52].

On Day 0, blood was collected for tests including antibody titers, total immunoglobulin isotypes, C-reactive protein (CRP), and retinol (the latter measured by ARUP laboratories, test code 0080525). To define vitamin A sufficiency/insufficiency and insufficiency/deficiency, we used the previously described cut-off values of 0.3 µg/mL and 0.2 µg/mL retinol, respectively, recognizing that vitamin cut-off values remain an ongoing topic of debate [10,45,46,50,51,52,53,54]. For bloodwork assays, the pre-vaccine measurements were from Day 0 or from the screening visit if Day 0 samples were unavailable. Participants returned to the clinic on Day 21 for additional bloodwork and nutrition evaluation.

### 2.2. Food Diary Acquisition and Analyses

On Day 0, a registered dietitian nutritionist (RDN) met with the parent/guardian to educate them on the proper procedure for recording a food diary. Food diary forms were provided. On day 21, the RDN met with the parent/guardian to complete a 24 h recall. The food diary forms were used to assist caregivers by helping them remember food intake details. A Nutrition Data System for Research (NDSR), a Windows-based dietary analysis program designed for the collection and analyses of 24 h dietary recalls, was utilized to analyze nutritional data.

### 2.3. Immune Assays

Pneumococcus-specific antibodies were tested by ARUP (*Streptococcus pneumoniae* antibodies, IgG, 23 serotypes, Test code 2005779). Pneumococcal serotypes represented in the ARUP assay included 1,2,3,4,5,6B, 7F,8 9N,9V, 10A, 11A, 12F,14,15B,17F,18C,19A,19F, 20, 22F, 23F, and 33F. The test was a quantitative multiplex chemiluminescent assay. Data values that were below or above the limits of detection (lower limit of detection, LLD, or upper limit of detection, ULD) were replaced with the respective limit values. At least a two-fold increase over background and/or an antibody score of at least 1.3 µg/mL after vaccination defined a ‘good responder’ per the manufacturer’s recommendations.

Antibody responses toward the hepatitis A vaccine were measured by ARUP (test code 0020591), yielding a plus/minus score. The test was a qualitative chemiluminescent assay.

### 2.4. Total Serum Immunoglobulin Isotype Analyses

Total serum immunoglobulin isotypes were measured with a bead-based multiplex immunoassay (Millipore Sigma, Billerica, MA, USA). A Luminex 200 Multiplexing Instrument with xPONENT software, Version 4.3 (Luminex, Austin, TX, USA) was used. Data were analyzed using Milliplex Analyst software, Version 5.2 Flex (Carlisle, MA, USA). Data values that were below or above the limits of detection were replaced with respective limit values.

### 2.5. CRP Assay

The CRP test was performed with a kit (Invitrogen Human C-Reactive Protein ELISA Kit Catalog KHA0031, Waltham, MA, USA) following the manufacturer’s instructions. Plates were read on a Molecular Devices Versa Max plate reader, and data were analyzed using SoftMax Pro 7.3 software.

### 2.6. Statistical Methods

Spearman’s rank correlation coefficients and *p*-values were computed using GraphPad Prism software (Version 10) or the rcorr function in the R package (version 4.3.0) Hmisc (Version 5.1.1). Results were not adjusted for multiple comparisons. Mann-Whitney tests were performed with GraphPad Prism software (Version 10). For R package analyses, binary variables were converted to numerical dummy variables prior to calculating the correlation coefficients. Missing values were handled by excluding pairs of variables that contained missing values during the correlation computation. Data values that were below or above the limits of detection were replaced with the respective limit values. The correlation matrix was generated utilizing the rcorr function in the Hmisc package and visualized through the corrplot function in the corrplot package (version 0.92).

## 3. Results

### 3.1. Vitamin A and D Levels in Young, Healthy Children in the PCVIT Study

Children 1–4 years of age inclusive were enrolled in PCVIT. Characteristics of the 20 children who were evaluable and who completed the study are shown in Table 1. All but three of the children were 1–2 years of age.

Of note, there was a pause in recruitment during the COVID-19 pandemic, yielding asymmetric participant characteristics. Older, black participants were more frequently enrolled before the pause (2019–2020) and younger, white participants were more frequently enrolled after the pause (2023–2024), contributing to an unintended positive correlation between black race and age. Participants immunized during 2019–2020 received the Prevnar 13 vaccine, whereas most participants immunized during 2023–2024 received Prevnar 20. Nonetheless, randomization and stratification upon enrollment ensured that the test group (supplemented with 10,000 IU vitamin A at the time of vaccination) and the control group were well balanced.

Retinol, RBP, and vitamin D levels were measured at baseline. As shown in Table 1, none of the tested participants were vitamin A deficient (using a cut-off of 0.2 µg/mL, 0.7 µM retinol for insufficiency/deficiency [45,46,53]. For all three factors (RBP, retinol, and vitamin D), the participants 2–4 years of age exhibited values below the group median.

Both vitamin D and retinol were negatively associated with age (*r* = −0.61, *p* = 0.0047 for vitamin D and *r* = −0.48, *p* = 0.038 for retinol). While RBP often correlates well with retinol and is often used as a surrogate [48], the correlation between RBP and retinol was not significant in this study.

**Figure 1:**
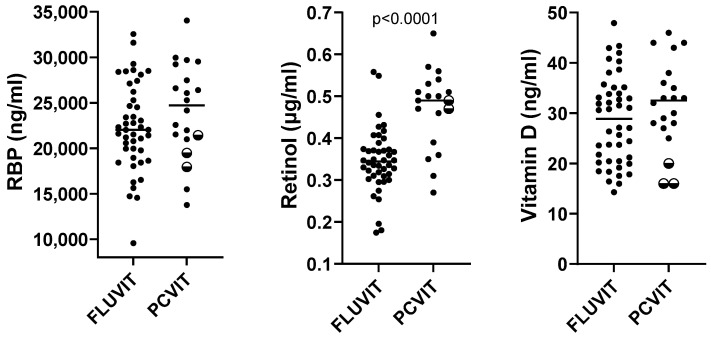
A comparison of baseline vitamin levels between participants in a previously described FLUVIT study and participants in PCVIT. The previously described FLUVIT study evaluated 44 children 2–8 years of age, enrolled during the years 2016–2017 [28]. Baseline vitamin levels are shown for comparisons between the FLUVIT and PCVIT study data. Each symbol represents a different study participant, with medians shown. There was a measurement for all 20 PCVIT study participants for RBP and vitamin D, and 19/20 participants for retinol. Two-toned symbols represent the three children 2–4 years of age in the PCVIT study; these values always fell at or below the group median. Mann–Whitney tests were performed to compare FLUVIT with PCVIT groups. The one significant *p* value is shown.

We compared PCVIT vitamin results (Table 1) with results from a previously performed study of healthy children 2–8 years of age living in Memphis, TN, USA. (FLUVIT 2016–2017 season, *n* = 44, 30% white, 70% black, [28]). Of note, the retinol assay was performed at a different location between the FLUVIT and PCVIT studies. Although both test sites measured unesterified retinol, there was a potential for assay differences. There was also a change in the version of the vitamin D assay during the study (Gen 3 replaced Gen 2 in 2022). As shown in Figure 1, children in the older FLUVIT population exhibited baseline values for RBP, retinol, and vitamin D that trended lower or were significantly lower than corresponding values for children in PCVIT.

### 3.2. Vitamin-Fortified Foods in Younger Children

To help explain the higher levels of vitamins among younger children, we considered that children 1–2 years of age may have had recent intakes of fortified formulas, milk, and/or baby foods. A requirement in the PCVIT study was that children were weaned from formula or breast milk for at least one month prior to study entry, but due to long half-lives, vitamins could have been sustained beyond one month from weaning.

An evaluation of food intakes among PCVIT participants provided further insights. Food intake data were available for twelve PCVIT study participants (Table 1). Most of these children had daily milk servings (naturally carrying vitamin A and presumably vitamin-fortified). There was a positive but non-significant correlation between milk servings and retinol levels (*r* = 0.51, *p* = 0.11). Among the twelve individuals with nutrition data, there were five (PCVIT014, 016, 017, 018, 019) with levels of retinol >0.3 µg/mL, RBP > 22,000 ng/mL, and vitamin D > 30 ng/mL. These five children were all less than 1.2 years of age, and in each case, milk servings were listed among food intakes. For four of the five participants (all but PCVIT014), baby food (presumably vitamin-fortified) was also listed in diets. Results support the hypothesis that frequent intakes of vitamin-rich and vitamin-fortified foods in children 1–2 years of age assisted maintenance of sufficient serum vitamin levels [55].

### 3.3. Pneumococcus Vaccine-Induced Immune Responses

For analyses of antibody responses toward the pneumococcus vaccine, focus was placed on the seven pneumococcal serotypes that were: (i) represented in both Prevnar 13 and Prevnar 20 vaccines, (ii) similarly immunogenic (within 5%) between Prevnar 13 and Prevnar 20 vaccines per the manufacturer’s package insert, and (iii) specifically tested in the ARUP assay. These were pneumococcal serotypes 5, 6B, 7F, 14, 18C,19A, and 19F. Results are shown in Table 2 for the antibody responses toward 19A and 19F pneumococcal serotypes, including measurements taken pre- and post-vaccination as well as the fold-change (Δ) between pre-vaccine and post-vaccine antibody values. It was found that 19A was the only pneumococcal serotype represented in the vaccine for which all pre-vaccine and post-vaccine antibody scores fell within the assay range, allowing for the best quantitative analyses of immune responses. In the 19A assay, all participants exhibited a ‘good response’ per the assay manufacturer’s instructions, in that participants showed at least a two-fold change in antibody levels between pre- and post-vaccination samples and/or they had a post-vaccination value of at least 1.3 µg/mL (Table 2).

Immune responses to the various pneumococcal serotypes represented in the vaccine were well correlated with one another. For example, the 19A-specific baseline (pre-vaccine) antibody levels correlated positively and significantly with pre-vaccine antibody levels against pneumococcal serotypes 5 (*r* = 0.51, *p* = 0.023), 7F (*r* = 0.45, *p* = 0.045), 14 (*r* = 0.46, *p* = 0.04), 18C (*r* = 0.48, *p* = 0.033), and 19F (*r* = 0.68, *p* = 0.001). The 19A-specific antibody Δ values correlated positively and significantly with Δ values for pneumococcal serotypes 5 (*r* = 0.72, *p* = 0.0003), 6B (*r* = 0.61, *p* = 0.004), 7F (*r* = 0.53, *p* = 0.016), 14 (*r* = 0.65, *p* = 0.002), 18C (*r* = 0.49, *p* = 0.028), and 19F (*r* = 0.65, *p* = 0.002). The participant with the lowest retinol level (PCVIT006, who had been randomized to the unsupplemented control group) exhibited the weakest Δ antibody responses against several pneumococcal serotypes represented in the vaccine (5, 6B, 14, 18C, 19A, and 19F).

A focus on the pneumococcal serotype 19F-specific antibody response in the PCVIT study identified additional correlations. Pre-vaccine antibodies specific for pneumococcal serotype 19F correlated positively with age (*r* = 0.45, *p* = 0.047) and negatively with Δ19F-specific antibody scores (*r* = −0.78, *p* < 0.001). Vitamin D was negatively correlated with pre-vaccine 19F-specific antibody scores (*r* = −0.5, *p* = 0.026) and age (*r* = −0.61, *p* = 0.0047), and was positively correlated with Δ19F-specific antibody scores (*r* = 0.45, *p* = 0.044). Black race was correlated positively with age (*r* = 0.53, *p* = 0.017) as described above, negatively with vitamin D (*r* = −0.77, *p* < 0.001), positively with pre-19F-specific antibody values (*r* = 0.63, *p* = 0.003), and negatively with Δ19F-specific antibody scores (*r* = −0.59, *p* = 0.006).

### 3.4. Hepatitis A Vaccine Immune Responses

Table 2 shows scores for antibody responses toward the primary hepatitis A vaccine. In this assay, vaccine-induced immune responses were identified as a conversion from negative to positive scores pre- and post-vaccination. For four participants, samples were unavailable or were positive prior to vaccination, perhaps due to a natural virus exposure. Among the remaining 16 participants, there were ten positive vaccine-induced immune responses detectable at the 21-day timepoint. The lack of responses in some participants was not surprising, as the hepatitis A vaccine was given as a primary dose, and follow-up tests were performed only 21 days post-vaccination [56]. The Δ response for hepatitis A-specific antibodies was not significantly associated with vitamin levels but was positively correlated with milk servings (*r* = 0.67, *p* = 0.035).

### 3.5. Influences of Supplementation in This Predominantly Retinol-Sufficient Pediatric Population

**Figure 2:**
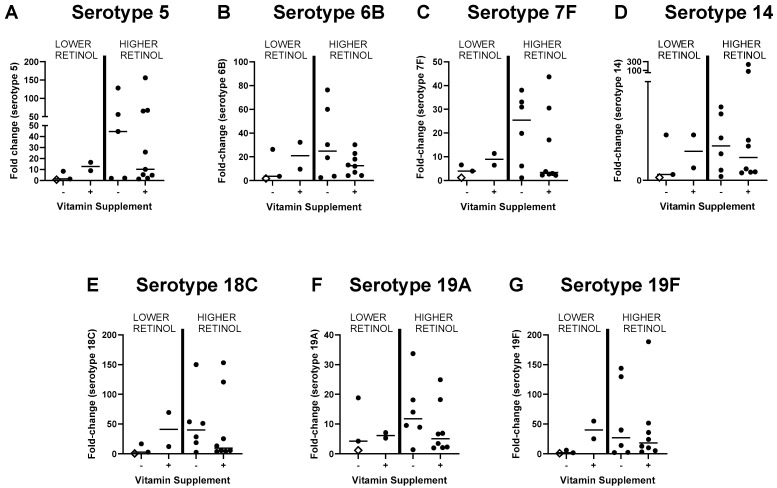
Antibody responses toward pneumococcal serotypes represented in the vaccine following Prevnar vaccinations with or without high-dose vitamin A supplements in a predominantly retinol-sufficient pediatric population. Graphs are labeled by the particular pneumococcal serotype (termed ‘serotype’) toward which antibodies responded. Each symbol represents a different participant. The x-axis identifies test (vitamin supplemented, +) and control (unsupplemented, −) participant groups. The y-axis identifies differences (fold-change, Δ) in specific antibody levels toward a given pneumococcal serotype represented in the vaccine, before and after vaccination. Participants were further grouped according to baseline vitamin levels (cut-off = 0.4 µg/mL retinol). Medians are shown for Δ antibody levels in each group. Only one participant (open diamond) had a baseline retinol level below 0.3 µg/mL. Among children with the higher retinol levels at baseline, median pneumococcus-specific antibody fold-change (Δ) levels trended lower in the supplemented group. In this figure, 19A was the only pneumococcal serotype represented in the vaccine for which all specific antibody values fell within assay range. The cut-off value for the limit of detection was used when pneumococcus-specific antibody values were out of range.

Figure 2 provides a graphic display of Δ values for antibodies specific for pneumococcal serotypes 5, 6B, 7F, 14, 18C, 19A, and 19F. Given that no children in the PCVIT study were considered vitamin A deficient (using 0.2 µg/mL retinol as the cut-off), participants were arbitrarily grouped using 0.4 µg/mL retinol as a cut-off. As shown in Figure 2, vitamin A supplementation did not improve immune responses toward the pneumococcal serotypes represented in vaccines in this predominantly retinol-sufficient population. Figure 2 further shows that the vitamin supplemented children with the higher baseline retinol values exhibited a negative trend in Δ antibody values compared to controls. There was also a trend toward a lower percentage of positive Δ antibody scores toward the hepatitis A vaccine among evaluable participants in the >0.4 µg/mL retinol category when vitamin supplemented participants were compared to unsupplemented, control participants (vitamin supplemented participants = 2 positive, 3 negative (40% positive); vitamin unsupplemented, control participants = 4 positive, 2 negative (67% positive)).

### 3.6. Total Immunoglobulin Isotypes and CRP Levels at Baseline

**Figure 3:**
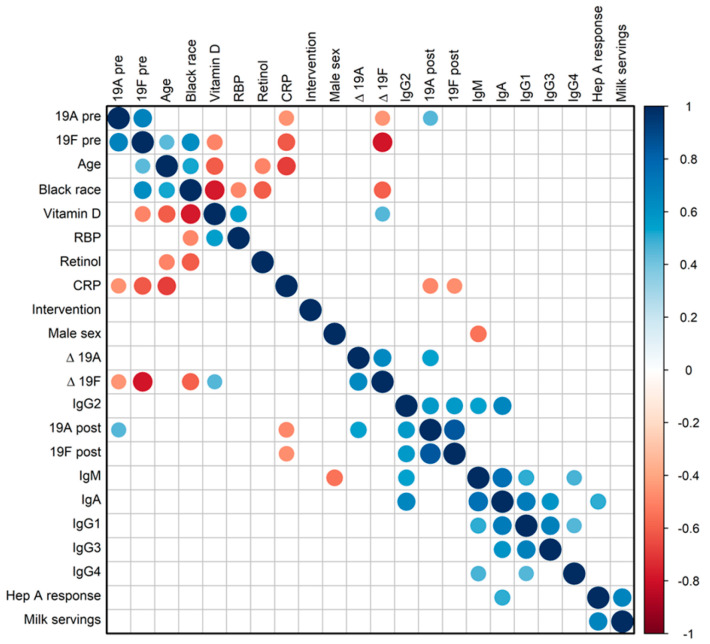
Correlation matrix analyses. In this correlation matrix, circle sizes represent the absolute values of the corresponding correlation coefficients, and circle colors indicate the +/− values of the correlation coefficients. The arrangement of parameters was determined using hierarchical clustering (‘hclust’). Two vaccine-induced antibody responses were included. These were specific for pneumococcal serotype 19A, for which no values were above or below the assay’s limit of detection, and specific for pneumococcal serotype 19F. Non-significant (*p* > 0.05) coefficients were not displayed.

To examine additional factors that might influence or be influenced by vitamin levels or immune responses [57,58,59,60,61,62,63,64,65,66,67], total serum immunoglobulin isotypes and CRP levels were measured at baseline (Table 3) and a full correlation matrix was constructed (Figure 3) including demographics, vitamin levels, immune responses, total immunoglobulin isotypes, and CRP. Antibody scores toward pneumococcal serotypes 19A and 19F (pre-vaccine, post-vaccine, and Δ) were used as representatives. Total IgG2 correlated positively with 19A and 19F post-vaccine antibody scores (*r* = 0.57, *p* = 0.008 for both). Total IgA correlated positively with the hepatits A-specific antibody Δ response (*r* = 0.5, *p* = 0.046). CRP correlated negatively with age (*r* = −0.69, *p* = 0.0008), pre-19A-specific antibody scores (*r* = −0.46, *p* = 0.042), post-19A-specific antibody scores (*r* = −0.49, *p* = 0.03), pre-19F-specific antibody scores (*r* = −0.61, *p* = 0.004), and post-19F-specific antibody scores (*r* = −0.47, *p* = 0.037).

## 4. Discussion

PCVIT was designed to determine if a high-dose, oral, vitamin A supplement would improve immune responses toward a booster pneumococcus vaccine and/or a primary hepatitis A vaccine in healthy 1–4 year old children living in Memphis, TN, USA. A total of 20 participants completed the study, all but three of whom were 1–2 years of age.

### 4.1. Vitamin Levels Among PCVIT Participants

Eighteen of the 19 participants in PCVIT who were tested for retinol had a value of at least 0.3 µg/mL and no participant was considered deficient (using a 0.2 µg/mL cut-off for deficiency) [53]. These vitamin levels were higher than those of older Memphian children and adults described in previous studies [24,28,68]. One explanation for the differences was indicated by food diaries. In the PCVIT study, there were frequent intakes by children 1–2 years of age of milk and baby foods that were likely vitamin-fortified [55]. A positive but insignificant correlation was observed between milk servings and retinol levels. Young children in the PCVIT study may have also retained retinol from previous formula feedings, even though they were required to have been weaned from formula or breastfeeding for at least one month prior to study enrollment. Unfortunately, a reduction in nutrition has been reported as a common occurrence as children age [69,70,71,72].

The PCVIT study differed from previous studies in that there was a negative correlation between retinol with age and a lack of correlation between RBP and retinol. RBP serves as a retinol escort and is often used as a surrogate for retinol levels [48]. However, RBP values can be influenced by a variety of factors, including season [68,73], metabolic disease [74,75], inflammation [48,73,74,75,76,77,78,79], and kidney disease [78,80].

### 4.2. Vaccine-Induced Immune Responses Among PCVIT Participants

Pre-vaccine, post-vaccine, and Δ antibody scores were evaluated against pneumococcal serotypes and hepatitis A. Most participants in the PCVIT study responded to vaccines with increased antibody levels. Antibody scores against the different pneumococcal serotypes were often positively correlated with one another. The one child with a retinol level at baseline of less than 0.3 µg/mL (who was randomized to the control arm of the study) had the worst Δ antibody response toward several pneumococcal serotypes. The Δ antibody response against hepatitis A was positively correlated with milk servings, and milk servings correlated positively, although not significantly, with retinol. These results, while observational, were consistent with previous findings suggesting that baseline vitamin A is beneficial to antibody responses [28]. Vitamins regulate cells both by internal and external interactions [2]. As an example, vitamins A and D are among nuclear receptor ligands with response elements situated in key regulatory regions of immunoglobulin loci [6,20,81].

A focus on the antibody responses to pneumococcal serotypes 19A and 19F represented in the vaccine revealed additional correlations. There was a negative correlation between pre-vaccine antibody scores and Δ antibody scores specific for 19F, a situation that is similar to that of previous vaccine studies [57,73,82]. The result may be due, at least in part, to enhanced vaccine clearance when there is a robust pre-vaccine immune response, thus dampening vaccine efficacy. Vitamin D and CRP were each associated negatively with age [83] and negatively with pneumococcus pre-vaccine antibody scores specific for pneumococcal serotype 19F. Black race was associated positively with age as described above, positively with the pre-vaccine antibody score specific for pneumococcal serotype 19F, and negatively with the Δ antibody score specific for pneumococcal serotype 19F. Total serum IgG2 at baseline correlated positively with post-vaccine antibody scores (specific for pneumococcal serotypes 19A and 19F) and total serum IgA correlated positively with the Δ antibody score specific for hepatitis A. IgG2 is known for its anti-carbohydrate function and its association with an Fc receptor that may assist antigen persistence and presentation [84,85,86]. IgA may similarly influence the trafficking and presentation of hepatitis A [87,88]. While these observational data help to generate hypotheses, additional research is warranted to prove cause–effect relationships and dissect the mechanisms responsible for vaccine-induced immune responses.

### 4.3. No Benefit of Vitamin A Supplementation Revealed Among Healthy 1–2 Year Old Children with Sufficient Levels of Retinol at Baseline

In the context of high baseline retinol levels, the vitamin A supplement did not appear to benefit the vaccine-induced antibody responses, either toward the pneumococcus or hepatitis A vaccine components. In fact, among children who had baseline levels of retinol above 0.4 µg/mL, there were downward trends of pneumococcus-specific antibody responses when vitamin supplemented children were compared to controls. Results are reminiscent of the FLUVIT study in which children received an influenza virus vaccine with or without vitamin A and D oral supplementation. In that case, vitamin supplements were only supportive of improved immune responses when children exhibited relatively low levels of RBP and vitamin D at baseline [28].

The PCVIT study confirms that while high-dose vitamin supplementation programs can be beneficial [26,27], the benefit does not necessarily translate to all populations [43,44]. Further clinical testing is encouraged before programs of high-dose vitamins are implemented in vitamin-sufficient pediatric populations.

### 4.4. Study Limitations

There were a number of limitations associated with the PCVIT study. The study was small (*n* = 20), was performed in only one location, and was designed to evaluate only one dose of oral vitamin A. The study was paused during the COVID-19 pandemic, yielding skewed participant characteristics with regard to age, race, and type of vaccination, each of which may have influenced outcomes. The version of the vitamin D assay changed over time. Measurements for antibody responses to the Prevnar vaccine and immunoglobulin isotypes often fell above or below the limits of detection. Analyses were not controlled for multiple comparisons. Further, antibody responses were only analyzed on day 21. A longer study may have revealed more information.

These limitations weakened our capacity to identify benefits of vitamin A supplementation. We instead observed that vitamin supplemented participants with relatively high baseline vitamin A levels trended toward lower vaccine-induced immune responses compared to unsupplemented controls. Data encourage the conduct of larger and longer studies to validate calls for vitamin supplementation in populations that are vitamin-sufficient.

## 5. Conclusions

Here, we observed that a population of predominantly 1–2 year-old children living in Memphis, TN, exhibited better serum vitamin levels compared to previously studied older children from the same geographical location. One explanation for the higher vitamin levels in children 1–2 years of age could have been recent or current intakes of vitamin-fortified formulas, milk, and baby foods. Age was associated positively with the pre-vaccine antibody scores specific for pneumococcal serotype 19F, and the pre-vaccine antibody scores associated negatively with the Δ antibody scores. Total serum IgG2 at baseline correlated with post-vaccine antibody scores specific for several pneumococcal serotypes, and total serum IgA at baseline correlated with hepatitis A-specific Δ antibody scores. We found no evidence in this predominantly retinol-sufficient population that an oral vitamin A supplement of 10,000 IU was beneficial to vaccine-induced immune responses. Results encourage further clinical research prior to the implementation of high-dose vitamin supplementation programs in pediatric, vitamin sufficient populations.

## Figures and Tables

**Table 1:** Participant characteristics.

PCVIT Participant	Sex	Race ^#^	Age (Years)	Enrollment Years *	Vaccine	Group ^&^	Retinol (µg/mL)	RBP (ng/mL)	Vitamin D (ng/mL)	Daily Milk Servings in Liquid Cup Equivalents
1	M	B	1.48	2019–2020	Prevnar 13	Control	0.31	26,066	29	0
2	M	B	1.48	2019–2020	Prevnar 13	Test	0.46	29,234	28	0
3	M	B	1.03	2019–2020	Prevnar 13	Test	0.39	15,492	36	NA
4	F	B	1.23	2019–2020	Prevnar 13	Test	0.36	34,045	33	NA
5	M	W	1.01	2019–2020	Prevnar 13	Control	0.54	29,702	44	NA
6	M	B	1.08	2019–2020	Prevnar 13	Control	0.27	24,149	30	NA
7	M	B	1.18	2019–2020	Prevnar 13	Test	0.49	22,568	32	NA
8	F	B	3.01	2019–2020	Prevnar 13	Test	0.47	21,425	20	NA
9	F	B	1.89	2019–2020	Prevnar 13	Control	0.35	21,514	33	NA
10	M	B	2.66	2019–2020	Prevnar 13	Control	0.49	19,480	16	NA
11	M	B	1.12	2023–2024	Prevnar 13	Test	0.50	13,755	25	2.79
12	M	B	1.22	2023–2024	Prevnar 20	Control	0.51	20,990	28	1.06
13	F	B	1.01	2023–2024	Prevnar 20	Control	0.56	27,472	27	0.06
14	M	W	1.11	2023–2024	Prevnar 20	Test	0.47	25,277	38	0.47
15	M	W	1.11	2023–2024	Prevnar 20	Test	0.51	21,986	33	0.47
16	M	W	1.08	2023–2024	Prevnar 20	Control	0.53	26,599	43	3.25
17	F	W	1.13	2023–2024	Prevnar 20	Test	0.57	29,952	46	1.16
18	F	W	1.04	2023–2024	Prevnar 20	Control	0.65	26,399	44	2.63
19	M	W	1.06	2023–2024	Prevnar 20	Test	0.50	29,526	35	2.39
20	F	B	2.70	2023–2024	Prevnar 20	Control	NA	17,931	16	2.39

Legend: Characteristics of the 20 participants who completed the PCVIT study are shown. ^#^ B = self-described as black or African American, W = self-described as white. * The study was paused between 2020 and 2023 due to the COVID-19 pandemic. ^&^ The test group received a vitamin A supplement, whereas the control group did not. Retinol, RBP, and vitamin D were tested at baseline. NA = not available. NDSR milk servings referred to liquid cup equivalents for milk drinks of any fat or no fat content, including regular, non-dairy, ready-to-drink, and powdered products (NDSR categories DMF0100, DMF0200, DML0100, DML0200, DML0300, DML0400, DML0500, DMN0100, DMR0100, DMR0200, MSC1100, MSC1300, SWT0600).

**Table 2:** Vaccine-induced immune responses.

PCVIT Participant	Pre-19A	Post-19A	Δ19A *	Pre-19F	Post-19F	Δ19F *	Pre-Hep A	Post-Hep A	Δ-Hep A
1	0.57	10.74	18.84 (+)	4.32	27.26	6.3 (+)	Negative	Negative	Negative
2	1.34	3.09	2.31 (+)	5.97	60.85	10.2 (+)	NA	NA	NA
3	2.03	10.75	5.30 (+)	3.8	96.6	25.4 (+)	Negative	Positive	Positive
4	0.34	2.41	7.09 (+)	0.4	21.95	54.9 (+)	Negative	Positive	Positive
5	1.07	14.96	13.98 (+)	0.87	112.96	129.8 (+)	Negative	Positive	Positive
6	2.05	2.44	1.19 (+)	15.48	15.19	1 (+)	Negative	Positive	Positive
7	0.52	1.1	2.12 (+)	1.51	5.12	3.4 (+)	NA	NA	NA
8	0.85	15.45	18.18 (+)	3.15	112.96	35.9 (+)	Positive	Positive	Unknown
9	4.96	21.17	4.27 (+)	58.55	112.96	1.9 (+)	Positive	Positive	Unknown
10	0.86	15.59	18.13 (+)	7.95	112.96	14.2 (+)	Negative	Positive	Positive
11	0.55	3.68	6.69 (+)	1.8	23.22	12.9 (+)	Negative	Positive	Positive
12	1.41	12.49	8.86 (+)	20.82	46.27	2.2 (+)	Negative	Negative	Negative
13	1.46	2.11	1.45 (+)	3.38	8.52	2.5 (+)	Negative	Negative	Negative
14	1.06	7.29	6.88 (+)	0.64	33	51.6 (+)	Negative	Positive	Positive
15	0.45	11.2	24.89 (+)	0.17	32.07	188.6 (+)	Negative	Negative	Negative
16	1.35	12.82	9.50 (+)	0.63	25.17	40 (+)	Negative	Positive	Positive
17	3.47	12.14	3.50 (+)	3.51	85.52	24.4 (+)	Negative	Negative	Negative
18	0.22	7.41	33.68 (+)	0.18	25.91	143.9 (+)	Negative	Positive	Positive
19	0.24	0.49	2.04 (+)	2.82	15.18	5.4 (+)	Negative	Negative	Negative
20	2.56	18.74	7.32 (+)	6.98	64.99	9.3 (+)	Negative	Positive	Positive

Legend. Pre-vaccine and post-vaccine antibody responses toward the pneumococcus and hepatitis A targets are shown. Pneumococcus-specific antibody scores are in µg/mL. * A ‘good responder’ to Prevnar vaccines per ARUP was a ≥ 2-fold improvement in antibodies from baseline or at least 1.3 µg/mL vaccine-specific antibodies at the post-vaccine visit. 19A and 19F-specific antibody levels are shown before (pre) and after (post) vaccination, along with differences (Δ). All 19A-specific antibody scores were within the limits of detection. For 19F-specific antibody scores, some exceeded 112.96 µg/mL, the upper limit of detection, influencing Δ values. NA = not available. The pre-vaccine and post-vaccine antibody responses to the hepatitis A vaccine were only scored as negative or positive. Ten individuals showed a positive Δ in that Hep A-specific antibody scores were negative prior to vaccination and positive after vaccination. Six individuals showed a negative Δ in that Hep A-specific antibody scores were negative both pre- and post-vaccination.

**Table 3:** Baseline total serum immunoglobulin isotypes and CRP in the study population.

PCVIT Participant	IgM (mg/mL)	IgG1 (mg/mL)	IgG2 (mg/ml)	IgG3 (mg/mL)	IgG4 (mg/mL)	IgA (mg/mL)	CRP (mg/L)
1	0.63	2.58	0.23	0.43	0.05	0.15	0.23
2	0.60	2.13	1.16	0.18	0.01	0.26	0.32
3	0.97	4.28	1.05	0.54	0.62	0.65	2.17
4	0.92	2.59	0.23	0.86	0.00	0.33	1.22
5	0.65	3.42	1.97	0.58	0.00	0.35	0.53
6	1.38	5.17	0.42	1.63	0.03	0.54	0.3
7	0.49	4.19	0.23	2.08	0.00	0.30	0.66
8	2.36	6.21	2.34	2.54	1.52	0.48	0.19
9	0.74	3.71	0.79	2.16	0.00	0.40	0.21
10	1.09	7.41	3.78	2.71	3.77	1.24	0.42
11	0.43	3.40	0.23	0.33	0.02	0.14	0.36
12	0.68	2.59	0.23	0.21	0.01	0.15	0.35
13	1.33	5.17	1.20	2.71	0.01	0.55	2.93
14	0.76	4.24	0.23	2.00	0.03	0.35	0.78
15	0.63	2.75	0.46	1.72	0.00	0.23	0.6
16	0.89	3.77	1.17	0.60	0.30	0.31	0.98
17	0.68	1.66	0.82	0.61	0.02	0.16	0.1
18	1.79	3.48	0.91	0.82	0.05	0.34	4.51
19	0.57	4.53	0.23	1.50	0.04	0.19	0.72
20	1.31	3.69	1.94	1.71	0.04	0.62	0.16

Legend. Total serum immunoglobulin isotypes (mg/mL) and CRP (mg/L) measurements are shown for each of the study participants. Limits of detection included 0.23 (LLD) for IgG2, 2.71 (ULD) for IgG3, and 3.77 (ULD) for IgG4.

## Data Availability

Raw data are available upon request to the authors.
